# High levels of plasma biomarkers at 24 h were found to be strong predictors of 90-day mortality: beware of some potential confounders!

**DOI:** 10.1186/s13613-021-00838-0

**Published:** 2021-03-15

**Authors:** Patrick M. Honore, Sebastien Redant, Thierry Preseau, Keitiane Kaefer, Leonel Barreto Gutierrez, Rachid Attou, Andrea Gallerani, David De Bels

**Affiliations:** 1 ICU Dept, Centre Hospitalier Universitaire Brugmann-Brugmann University Hospital, Place Van Gehuchtenplein, 4, 1020 Brussels, Belgium; 2 ED Dept, Centre Hospitalier Universitaire Brugmann, Brussels, Belgium

We have read with great interest the recently published paper by Jäntt et al. who conclude that in their study high levels of both plasma proenkephalin (P-PENK) and plasma neutrophil gelatinase-associated lipocalin (P-NGAL) at 24 h were found to be strong and independent predictors of 90-day mortality [1]. We would like to comment. When looking carefully in the study data, acute kidney injury (AKI) stage 1B was present in 42% of the patients. In addition, supplemental patients went into AKI by 12 h (almost 20%) [1]. So, this means at least more than 60% were in AKI by 12 h [1]. It stands to reason that some of those patients were put under renal replacement therapy (RRT) and even continuous RRT (CRRT) by the first 12 to 24 h. The incidence of RRT in the study was 14% but although we do not know when they started, it is highly probable that they start quite early. Indeed seeing the severity of these cardiogenic patients and the high incidence of AKI at baseline and at 12 h, clearly some patients were upon RRT or CRRT (seeing the high prevalence of vasopressors) within the first 12 to 24 h [1]. P-NGAL is a secretory protein whose molecular weight (MW) is 25,000 dalton (Da) as a monomer [2]. P-PENK has a MW of 4586 Da [2]. The contemporary CRRT membranes are able to remove molecules as large as 35,000 Da [3]. Hence, both P-PENK and P-NGAL could easily be removed by CRRT [3]. When new highly adsorptive membranes (HAM) with high absorptive abilities are used, the ability of CRRT to eliminate both P-PENK and P-NGAL could be even enhanced [4]. Accordingly, if P-PENK and P-NGAL are used to predict 90-day mortality in those patients, falsely low both P-PENK and P-NGAL in CRRT patients could lead a wrong and falsely lower prediction of 90-day mortality. If at some stage, both P-PENK and P-NGAL are used for de-escalation in the future, falsely low P-PENK and P-NGAL levels in CRRT patients, in turn, could lead to an earlier de-escalation of support therapy. There has been no investigation on the performance of P-PENK on patients who receive CRRT. Therefore, we believe there is a critical need for a future study with a focus on the performance of the currently known sepsis biomarkers among those who receive CRRT [5].

## Data Availability

Not applicable.

## Abbreviations

P-PENK: Plasma proenkephalin
P-NGAL: Plasma neutrophil gelatinase-associated lipocalin
AKI: Acute kidney injury
RRT: Renal replacement therapy
CRRT: Continuous renal replacement therapy
HAM: Highly adsorptive membranes
